# Exploring the effect of pain on response to reward loss in calves

**DOI:** 10.1038/s41598-023-42740-8

**Published:** 2023-09-16

**Authors:** Thomas Ede, Marina A. G. von Keyserlingk, Daniel M. Weary

**Affiliations:** 1 Animal Welfare Program, Faculty of Land and Food Systems, University of British Columbia, 2357 Main Mall, Vancouver, BC V6T 1Z6 Canada; 2 Department of Clinical Studies, Swine Teaching and Research Center, University of Pennsylvania School of Veterinary Medicine, Kennett Square, PA USA

**Keywords:** Animal behaviour, Zoology

## Abstract

Negative emotional states are known to interact, potentially aggravating one another. In this study, we used a well validated paradigm (successive negative contrast, SNC) to determine if pain from a common procedure (disbudding) influences responses to a reward downshift. Holstein calves (n = 30) were trained to approach a 0.5 L milk reward. Latency to approach, number of vocalisations and pressure applied on the bottle were recorded during training. To assess how pain affected responses to reward downshift, calves were randomly assigned to one of three treatments before the downshift. Two groups were disbudded and provided the ‘gold standard’ of pain mitigation: intraoperative local anesthesia and analgesia. One of these disbudded groups was then provided supplemental analgesic before testing. The third group was sham disbudded. All calves were then subjected to the reward downshift by reducing the milk reward to just 0.1 L. Interactions were detected between test session and daily trials on pressure applied for the Disbudded group (estimate ± SEM: 0.08 ± 0.05), and on vocalisations for the Sham (0.3 ± 0.1) and Disbudding + Analgesia (0.4 ± 0.1) groups. Our results indicate that SNC is a promising paradigm for measuring negative affect in calves and suggests that pain potentially affects the response to a reward downshift.

## Introduction

A large body of research, primarily on rodents, has shown that sudden declines in reward levels are highly salient and provoke a negative affective response consistent with feelings of frustration or disappointment^1,2^. A well-developed paradigm for provoking this response is the successive negative contrast (SNC) test, where animals learn to obtain feed rewards which are then reduced in quantity or quality. Multiple lines of evidence indicate that this experience is distressing for animals, including increased levels of physiological markers of stress in rats and pigs^3–5^, and development of a preference for anxiolytic medication in rats^6^. In addition, responses to SNC are aggravated when an animal is in a pre-existing negative emotional state at the time of the test. For example, rats bred to be more anxious had higher latencies to approach a reward after a downshift^7^, and rats in amphetamine withdrawal displayed greater and longer reductions in reward consumption following a downshift^8^.

The influence of current affective state on SNC responses provides a compelling opportunity for the assessment of animal welfare, although only a handful of studies have employed this approach. In one study, rats housed in barren environments showed an extended increase in latency to approach the downshifted reward in comparison to rats housed in enriched environments, suggesting that these animals were more sensitive to reward loss than were rats in enriched housing^9^. Housing conditions (barren vs. enriched) also affected pigs’ sensitivity to reward loss^10^. To our knowledge, SNC has not been used to assess the emotional impact of pain in any species.

Research conducted on humans report that patients in a negative emotional state are more inclined to show anger responses^11,12^, and that pain can aggravate frustrating situations^13^.

In this study we tested if pain aggravates responses to SNC testing. Young cattle experience pain associated with routine farm procedures including hot-iron disbudding, indicated by physiological, behavioral and emotional responses to the procedure^14–18^. In this study we assessed the responses to SNC (in this case reducing the amount of milk available) in calves for three days following disbudding. Although providing a combination of local anesthesia and analgesia is considered a gold-standard in pain mitigation following disbudding, the duration of pain control has been challenging to estimate^18^, and disbudding pain has been suggested to last for several days^19–21^. For ethical reasons, all disbudded calves were provided local anesthesia and analgesia at the time of the procedure. To explore the potential longer-term pain caused by disbudding, a group of calves were provided additional fast-acting analgesia before tests. We predicted that calves in pain would respond to the downshift by increased pressure applied on the bottle containing the milk, number of vocalisations and latency to approach the reward. By exploring a novel approach to assessing the affective component of pain in animals, we hope to further the understanding of the emotional impact of a common farm procedure and, more generally, how negative states can interact to influence animal welfare.

## Results

**Figure 1:**
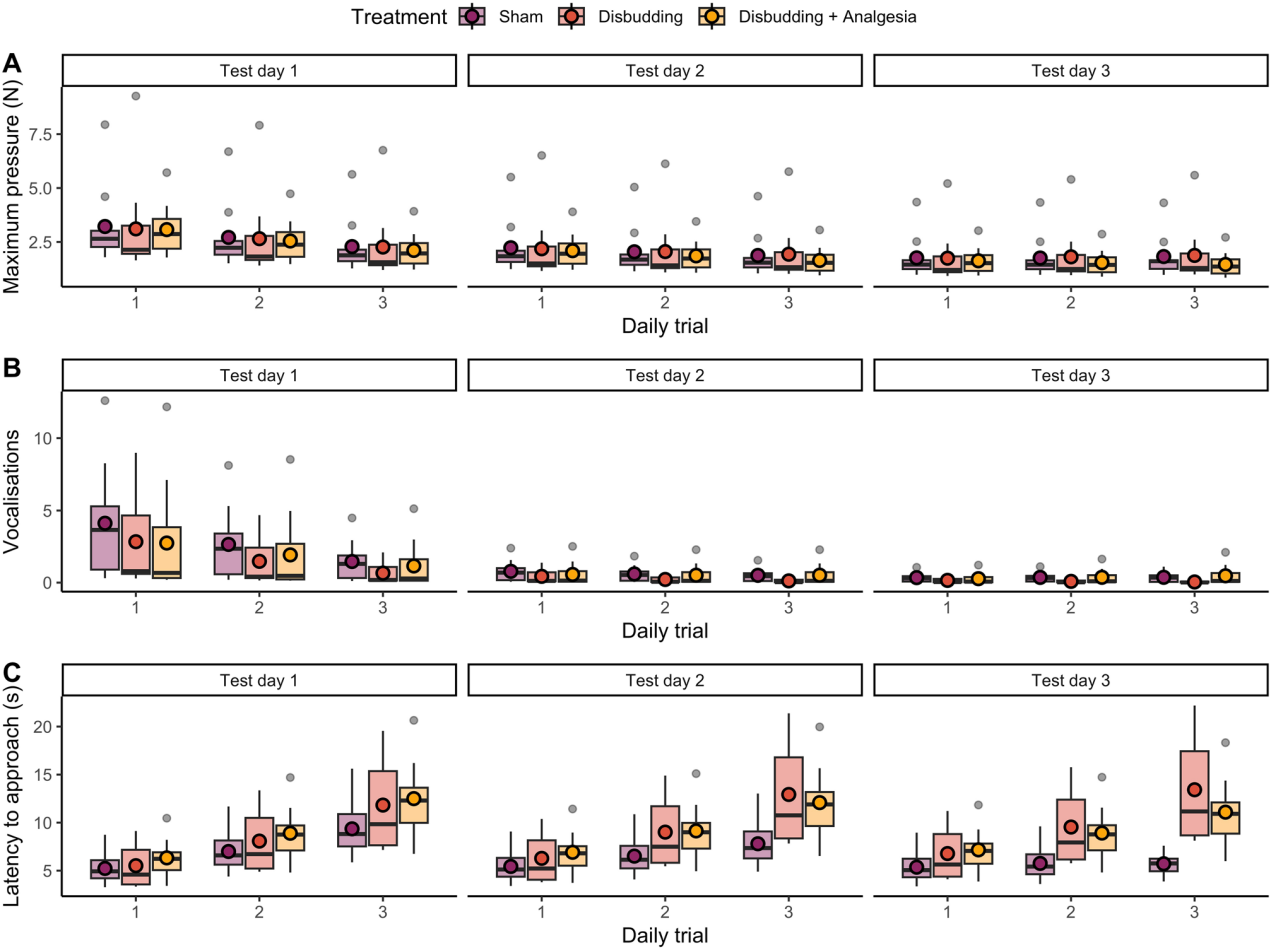
Calf responses to a reward downshift (from 0.5 L of milk to 0.1 L) over 3 test days, with 3 trials each day. An algometer was mounted behind the bottle containing the reward, measuring the maximum pressure exerted on the bottle by the calf (**A**). Vocalisations (**B**) and latency to approach (**C**) were also recorded. 24 h before the first test, calves were disbudded (Disbudding, Disbudding + Analgesia) or received a sham disbudding (Sham). One hour prior to tests, calves from the Disbudding + Analgesia group were administered an additional fast acting NSAID. Values presented are back transformed predicted values from mixed models. Colored circles represent mean estimates.

Maximum pressure applied to the reward bottle decreased across test days (− 0.4, t = − 3.5, *P* = 0.005) and daily trials (− 0.3, t = − 2.6, *P* = 0.01) (Fig. 1A). We also noted an interaction between test day and daily trial for the Disbudded group (0.1, t = 2.0, *P* = 0.05), and a tendency for the Sham group (0.08, t = 1.7, *P* = 0.09). There was no evidence of an interaction for pressure in the Disbudding + Analgesia group (0.07, t = 1.4, *P* = 0.2). Calves produced fewer vocalisations across days (− 1.5, t = − 4.6, *P* < 0.001) and daily trials (− 0.8, t = − 3.6, *P* < 0.001) (Fig. 1B). However, there were positive interactions between test day and trials for the Sham and Disbudding + Analgesia groups (0.3, t = 2.0, *P* = 0.05; 0.4, t = 2.7, *P* < 0.001 respectively). No interaction was found for the Disbudding group (0.05, t = 0.3, P = 0.7). Calves took longer to approach the reward across daily trials (0.4, t = 3.1, *P* = 0.002), with no effect of the test day (0.12, t = 0.9, *P* = 0.4) (Fig. 1C). Calves from the Sham group tended to decrease their latency across test day and daily trial (− 0.11, t = − 1.7, *P* = 0.09), whereas no interaction was found for the Disbudding (− 0.02, t = − 0.3, *P* = 0.8) and Disbudding + Analgesia groups (− 0.06, t = − 0.9, *P* = 0.3).

## Discussion

After the reward downshift, calves responded by high pressure applied to the bottle and vocalisations. As calves went through more test sessions (with the lower reward), vocalisations and pressure both decreased, suggesting calves updated their expectations over time. Following the downshift, calves also increased their approach latency across trials within daily test sessions. This result is consistent with previous reports noting that approach latency increased after reward downshift ^9,22^.

We found some indication of a treatment effect on responses to the downshift over test days and daily trials. The significant positive interaction in pressure applied on the bottle over days and trials for the *Disbudding* group suggests some level of maintained frustration over tests. Similarly, only calves who had not been disbudded tended to decrease their approach latency across trials whereas calves from the *Disbudding* and *Disbudding* + *Analgesia* groups maintained their latency increase. This result is consistent with results from Burman and colleagues^9^ who reported a more prolonged response to reward downshift (i.e. higher latency) from rats assumed to be in a more negative affective state. This result is also consistent with work on calves showing increased anticipatory behavior in response to a reward downshift for animals housed in a more barren environment^23^. We had expected that calves receiving supplemental ketoprofen before the daily test sessions would have responded similarly to the sham calves. That these animals appeared to have some similar responses to the other disbudded calves suggests that our ketoprofen treatment protocol might not have mitigated the pain associated with disbudding during tests. Ketoprofen has been noted as appropriate pain control for disbudding^24–27^, but conflicting results have also been reported^28,29^.

In a study on SNC in chickens, Davies and colleagues^30^ found a gradual increase in approach latency and an immediate response in consummatory behaviours. They noted the gradual increase to be consistent with Thorndike’s law of effect^31^, analogous to an extinction mechanism where a less valuable reward induces a less ‘enthusiastic’ response over time. The disparity with consummatory responses was suggested to relate to the different timeframes of the measures: anticipatory responses such as approach latency might require conditioned learning, and therefore change more slowly. However, consummatory responses such as pressure applied are immediate indicators of reward evaluation, and therefore do not require an adjustment delay.

Contrary to our predictions, calves from the *Disbudding* group did not vocalize more than the other treatment groups after the downshift. These results remain unclear to us, but the very low number of vocalisations past the first test day questions the sensitivity of calves vocalisations counts when used in SNC paradigms.

The high variability among calves in their response to the downshift could be associated to intrinsic individual differences. Individual differences in traits such as fearfulness have been linked to pessimistic responses to a judgment bias test^32^. Moreover, such pessimism was also linked to the anhedonic response (i.e. a decrease in interest in a consummatory reward) following hot-iron disbudding^33^. Calves’ individual differences could also be dependent on the severity of the sensitization of their head caused by the procedure^34,35^, causing increased pain when coming into contact with the bottle. Alternatively, sucking on the nipple (even without milk) may be positive for calves^36^ and may also provide pain relief in the hours after disbudding^37^.

## Conclusion

Following a reduction in a milk reward, calves who experienced a painful procedure appeared to potentially display an extended response to the downshift. Although SNC seems a promising avenue, our results remain tentative and further development of the paradigm and its applications must be investigated to identify its relevance to animal welfare assessment.

## Methods

### Ethics statement

Procedures were approved by The University of British Columbia Animal Care Committee under application A21-0111 and conducted in accordance with guidelines form the Canadian Council of Animal Care^38^. Reporting followed ARRIVE guidelines.

### Animals and housing

The study was conducted at The University of British Columbia's Dairy Education and Research Centre. To our knowledge, no study has used a similar paradigm in calves. To establish a sample size estimate, we relied on welfare studies using analogous SNC paradigms but applied to other species: rats (six subject per treatment^9^) and pigs (sixteen subjects per treatment group^10^). Considering this range and our own practical limitations, we settled on a sample size of ten subjects per treatment group. Thirty-five Holstein calves (all females) were initially enrolled in the study. Five calves were removed from the trial: three fell ill (scours and fever), one showed an extreme stress response when moved outside of her home pen, and one was not feed-restricted before a test. The thirty remaining had an average (± SD) birth weight of 38.3 ± 4.1 kg and were enrolled at 39.9 ± 4.1 d of age.

As routine farm practice, calves from all three treatments were intermingled in indoor pens (4.9 × 7.3 m, bedded with sawdust, and each containing eight to ten calves). Calves were provided ad libitum access to water and hay (RIC; Insentec B.V., Netherlands), and time-restricted access to 12 L of whole milk through a nipple feeder (CF 1000 CS Combi; DeLaval Inc., Sweden). To avoid long delay during trials, small replicates (average number of subjects per replicate = 3.5) were conducted.

### Apparatus

**Figure 2:**
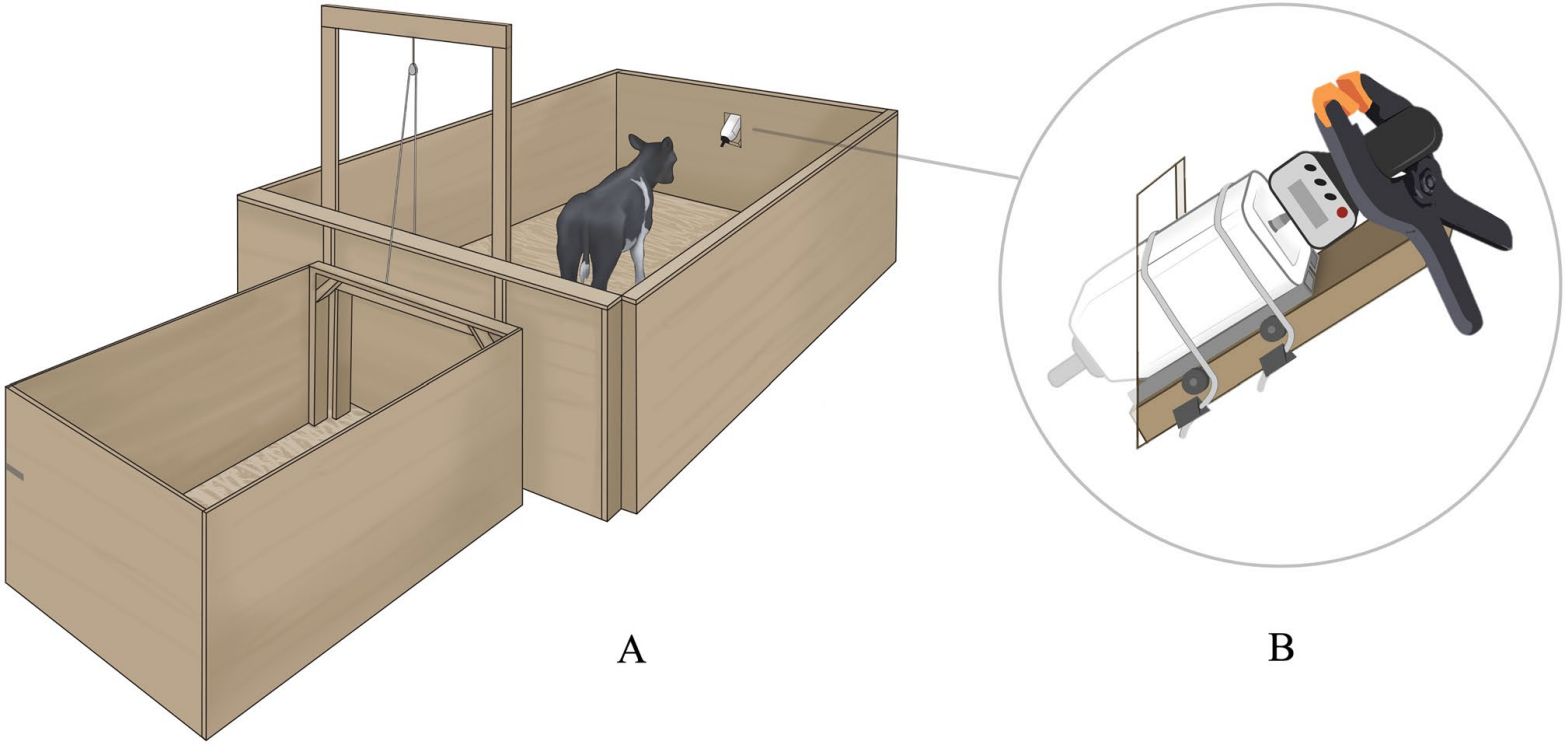
Calves were brought to the start-box, the vertical gate was lifted, and calves could access a milk reward (0.5 L during training, 0.1 L during tests) in the test pen (**A**). The bottle containing the milk reward was mounted on rails with an algometer positioned behind the bottle to measure the maximum pressure applied by the calf (**B**). Illustrations by Ann Sanderson.

The experimental apparatus was located in the same barn as the calves’ home pen, approximately 10–30 m away. The apparatus was a 1.8 × 1.2 m start-box leading to a 3.6 × 2.4 m pen through a vertical gate (Fig. 2A). Directly across from the start-box was a bottle and rubber teat mounted on rails, with an algometer (FPX 25, Wagner, Greenwich, USA) installed behind the bottle allowing measures of the maximum pressure applied to the bottle (Fig. 2B).

### Training

The trial was divided in three phases over seven days: training (three days), treatment (one day) and testing (three days). During training, calves were feed-restricted overnight (from 22:00 h) to ensure a high motivation for milk rewards over repeated trials. At approximately 10:00 h calves were individually brought into the apparatus, with no set order, and then placed in the start-box. The vertical gate was lifted and calves could approach and drink a 0.5 L milk reward from the bottle (this amount was based on previous studies on motivation trade-offs studies in calves^37,39^). Latency to contact the bottle (with mouth or tongue), latency to finish the reward, number of vocalisations and maximum pressure applied to the bottle were recorded live. The calf was then brought back to the start-box, the bottle refilled, and two more trials were conducted (i.e., for a total of three trials/d). After these trials were completed, the calf was returned to her home pen with full access to her daily milk allowance of (12 L/d). Training took place over three consecutive days, for a total of nine training trials. During the first day of training (for all three trials), no cues were given to the calf for the first minute after opening the start-box gate. After one minute, auditory (calls/whistle) and tactile (finger suckling) cues were given from the experimenter from outside the test-pen to get the calf’s attention towards the bottle. If these cues had failed after an additional minute, the experimenter would go inside the test pen and lead the calf to the bottle.

During the second and third day of training, no cues were given. If the calf had not approached the bottle within two minutes, the trial was recorded as a no-approach (and a pressure of zero applied to the bottle). Once a calf had approached the bottle, she had three additional minutes to finish the reward.

### Treatments

Calves were pseudo-randomly assigned to one of three treatments (*Disbudding*, *Disbudding* + *Analgesia, or Sham*; ten calves each). Treatment assignment was balanced for age and birthweight (*Disbudding:* 40.7 ± 4.3 d, 38.7 ± 3.9 kg; *Disbudding* + *Analgesia:* 39.2 ± 7.0 d, 38.0 ± 5.6 kg; *Sham:* 39.7 ± 6.0 d, 38.3 ± 2.4 kg). On treatment day, calves were not feed-restricted and went through their treatment in their group pen at approximately 10:00 h. Regardless of treatment, calves were weighted and administered a multimodal pain mitigation strategy of sedative, local anesthesia and analgesia. The sedative was used to facilitate following injections and disbudding (xylazine 0.2 mg/kg Subcutaneous, Rompun 20 mg/mL, Bayer, Leverkusen, Germany). After sedation was reached (recumbency and eye rotation, approximately 10 min), a local anesthetic was injected as a cornual nerve block to mitigate the acute pain of the procedure (5 mL per side, lidocaine 2%, epinephrine 1:100,000, Lido-2, Rafter8, Calgary, AB, Canada), an NSAID was provided to minimize inflammation (meloxicam 0.5 mg/kg Subcutaneous, Metacam 20 mg/mL, Boehringer Ingelheim, Burlington, ON, Canada), and the horn bud area was shaved with an electric trimmer. Ten minutes after lidocaine injection, a pinprick test was done on the horn buds to test for pain reflex. For calves in the *Disbudding* and *Disbudding* + *Analgesia* treatments, a pre-heated electric dehorner (X30, 1.3 cm tip, Rhinehart, Spencerville, IN, USA) was applied to both horn buds until a consistent dark ring formed around each bud (requiring approximately 10 to 15 s). Calves from the *Sham* group were treated identically but instead of being disbudded, only pressure on the horn buds was applied with the plastic handle of the dehorner. After the procedure was completed, calves were positioned in sternal recumbency and left to recover in the pen. As the magnitude and duration of NSAID effects following disbudding remain unclear ^18^, calves from the *Disbudding* + *Analgesia* group received an additional NSAID injection (ketoprofen, 3 mg/kg, Subcutaneous, Anafen, 100 mg/mL, Boehringer Ingelheim, Ontario, Canada) 1 h before each of the three test sessions to provide supplemental pain control at the time of testing. Based on a previous study on the efficacy of ketoprofen after disbudding^29^, we expected ketoprofen to provide analgesic effects for up to 2 h following treatment.

### Tests

In the three days following treatment, calves were tested for sensitivity to reward loss. Tests were similar to training: calves were brought individually to the apparatus after overnight feed restriction, and allowed access to a milk reward three times in a row (for a total of nine trials), but during testing the reward was reduced to 0.1 L. The time allowed for calves to approach and drink the reward was matched with their performance during training. Maximum pressure applied to the bottle, number of vocalisations and latency to approach were recorded. Calves from the *Disbudding* + *Analgesia* group received an additional NSAID injection (ketoprofen, 3 mg/kg, Subcutaneous, Anafen, 100 mg/mL, Boehringer Ingelheim, Ontario, Canada) 1 h before each of the three test sessions. After each session calves were returned to their home pen and again provided access to their full milk allowance (12 L). After the three test days calves were returned to routine farm care.

### Statistical analysis

A mixed model was conducted on each outcome (maximum pressure, vocalisations and approach latency) on test phases (post treatment) using R’s lme4 package^40^. For pressure and latency, data were log transformed to fit model assumptions of linearity, normality and homoscedasticity. For vocalisation counts, we used a Poisson mixed model. Fixed factors were treatment (2 df), test day (1 df), daily trial (1 df) and their interaction (3 df). Daily trial, nested within day and Calf ID, was included as a random factor. Significance and tendency thresholds were set at *P* ≤ 0.05 and *P* ≤ 0.10, respectively. Data (Supplementary Information 1) and R code (Supplementary Information 2) are available in supplementary materials.

### Supplementary Information

Supplementary Information 1.

Supplementary Information 2.

## Data Availability

The dataset and R code are freely available in supplementary materials.
