# Supplementary material for: Exploring the effect of pain on response to reward loss in calves
Source: Sci Rep. 2023 Sep 16;13:15403. doi: 10.1038/s41598-023-42740-8 (PMC10505155; doi:10.1038/s41598-023-42740-8)
Supplement: Supplementary file 2 — Supplementary Information 2. [file 41598_2023_42740_MOESM2_ESM.docx]

#data handling----

rm(list = ls())

library(ggplot2)

library(lme4)

library(lmerTest)

library(car)

library(viridis)

library(broom)

library(lmtest)

library(plyr)

library(ggpubr)

library(reshape)

data = read.csv("/Users/thomasede/Desktop/Research/Frustration pain/Writing/Scientific reports/R1/Revised analysis and plots/data.csv")

attach(data)

#data$pre_post = ifelse(Session_n <= 9, "Pre","Post")

data$Session <- factor(data$Session, levels = c("pre1","pre2","pre3","post1","post2","post3"))

data$Trt.Grp <- factor(data$Trt.Grp, levels = c("S","D","DA"))

attach(data)

data_post = subset(na.omit(data), Session == 'post1'|Session == 'post2'|Session == 'post3')

data_plot = subset(data, Session == "pre3"|Session == 'post1'|Session == 'post2'|Session == 'post3')

attach(data_plot)

data_plot$Session_plot = ifelse(Session == 'post1', 'Test day 1',

ifelse(Session == 'post2', 'Test day 2',

ifelse(Session == 'post3', 'Test day 3',

ifelse(Session == 'pre3', 'Training', 'wrong'))))

data_plot$Session_plot <- factor(data_plot$Session_plot, levels = c("Training","Test day 1","Test day 2","Test day 3"))

attach(data_post)

data_post$Session_n = ifelse(Session == 'post1', 1,

ifelse(Session == 'post2', 2,

ifelse(Session == 'post3', 3, 'wrong')))

attach(data_post)

#Revised analysis----

data_post$Session = ifelse(Session_n == 1, "Test day 1",

ifelse(Session_n == 2, "Test day 2",

ifelse(Session_n == 3, "Test day 3", "wrong")))

#pressure----

attach(data_post)

lm_p = lmer(data = data_post, log(Max_Pressure+1) ~ Trt.Grp + as.numeric(Session_n) + as.numeric(Round) + (Trt.Grp : as.numeric(Session_n) : as.numeric(Round)) + (1|Session_n/Round) + (1|ID))

summary(lm_p)

data_post$pred_p = predict(lm_p)

attach(data_post)

plot_p_pred = ggplot(data = data_post, aes(y = exp(pred_p), fill = Trt.Grp))+

geom_boxplot(aes(x = factor(Round)), alpha = .5, col = "grey15")+

facet_wrap(vars(Session), nrow = 1)+

stat_summary(aes(x = Round, fill = Trt.Grp), fun=mean, shape=21, size=0.6, color="black", position = position_dodge(width = 0.75)) +

scale_color_viridis(discrete = T, option = 'B', begin = 0.4, end = 0.8, direction = 1, name = 'Treatment', labels = c('Sham', 'Disbudding', 'Disbudding + Analgesia'))+

scale_fill_viridis(discrete = T, option = 'B', begin = 0.4, end = 0.8, direction = 1, name = 'Treatment', labels = c('Sham', 'Disbudding', 'Disbudding + Analgesia'))+

ylab("Maximum pressure (N)")+

xlab("Daily trial")+

theme_classic()

plot_p_pred

#vocalisations----

attach(data_post)

lm_voc = glmer(data = data_post, Vocalisations ~ Trt.Grp + as.numeric(Session_n) + as.numeric(Round) + (Trt.Grp : as.numeric(Session_n) : as.numeric(Round)) + (1|Session_n/Round) + (1|ID), family = poisson, control=glmerControl(optimizer="bobyqa",optCtrl=list(maxfun=2e5)))

summary(lm_voc)

data_post$pred_voc = predict(lm_voc)

attach(data_post)

plot_voc_pred = ggplot(data = data_post, aes(y = exp(pred_voc), fill = Trt.Grp))+

geom_boxplot(aes(x = factor(Round)), alpha = .5, col = "grey15")+

facet_wrap(vars(Session), nrow = 1)+

stat_summary(aes(x = Round, fill = Trt.Grp), fun=mean, shape=21, size=0.6, color="black", position = position_dodge(width = 0.75)) +

scale_color_viridis(discrete = T, option = 'B', begin = 0.4, end = 0.8, direction = 1, name = 'Treatment', labels = c('Sham', 'Disbudding', 'Disbudding + Analgesia'))+

scale_fill_viridis(discrete = T, option = 'B', begin = 0.4, end = 0.8, direction = 1, name = 'Treatment', labels = c('Sham', 'Disbudding', 'Disbudding + Analgesia'))+

ylab("Vocalisations")+

xlab("Daily trial")+

theme_classic()

plot_voc_pred

#latency----

attach(data_post)

lm_lat = lmer(data = data_post, log(Lat_App) ~ Trt.Grp + as.numeric(Session_n) + as.numeric(Round) + (Trt.Grp : as.numeric(Session_n) : as.numeric(Round)) + (1|Session_n/Round) + (1|ID))

summary(lm_lat)

data_post$pred_lat = predict(lm_lat)

attach(data_post)

plot_lat_pred = ggplot(data = data_post, aes(y = exp(pred_lat), fill = Trt.Grp))+

geom_boxplot(aes(x = factor(Round)), alpha = .5, col = "grey15")+

facet_wrap(vars(Session), nrow = 1)+

stat_summary(aes(x = Round, fill = Trt.Grp), fun=mean, shape=21, size=0.6, color="black", position = position_dodge(width = 0.75)) +

scale_color_viridis(discrete = T, option = 'B', begin = 0.4, end = 0.8, direction = 1, name = 'Treatment', labels = c('Sham', 'Disbudding', 'Disbudding + Analgesia'))+

scale_fill_viridis(discrete = T, option = 'B', begin = 0.4, end = 0.8, direction = 1, name = 'Treatment', labels = c('Sham', 'Disbudding', 'Disbudding + Analgesia'))+

ylab("Latency to approach (s)")+

xlab("Daily trial")+

theme_classic()

plot_lat_pred
